# Association between HMGA1 and immunosuppression in hepatocellular carcinoma: A comprehensive bioinformatics analysis

**DOI:** 10.1097/MD.0000000000032707

**Published:** 2023-01-27

**Authors:** Jie Zhu, Yongshun Zheng, Yuyao Liu, Mengding Chen, Yanyan Liu, Jiabin Li

**Affiliations:** a Department of Infectious Diseases, The First Affiliated Hospital of Anhui Medical University, Hefei, Anhui, China; b Department of General Surgery, The First Affiliated Hospital of Anhui Medical University, Hefei, Anhui, China; c Department of Burn, The First Affiliated Hospital of Anhui Medical University, Hefei, Anhui, China; d Anhui Center for Surveillance of Bacterial Resistance, Hefei, Anhui, China; e Institute of Bacterial Resistance, Anhui Medical University, Hefei, Anhui, China.

**Keywords:** biomarker, hepatocellular carcinoma, HMGA1, immunosuppression, prognosis

## Abstract

The high mobility group A1 (HMGA1) gene is overexpressed in malignant tumors, and its expression level correlates with the progression and metastasis of tumors. However, the specific role of HMGA1 in hepatocellular carcinoma (HCC) and relevant influencing approaches in tumor immunity remain unclear. In this study, the expression and clinical significance of HMGA1 in HCC immunity were analyzed. The expression levels of HMGA1 mRNA and protein in HCC tissue and normal liver tissue were analyzed based on the cancer genome atlas, the gene expression omnibus and the Human Protein Atlas databases. The correlation between HMGA1 and clinicopathological factors was analyzed, and survival was estimated based on the expression of HMGA1. Gene set cancer analysis and the TISIDB database were used to identify tumor-infiltrating immune cells and immune inhibitors. Gene set enrichment analysis was performed to determine the involved signaling pathway. The HMGA1 genetic alterations were identified with the cBioPortal for Cancer Genomics. The expression of HMGA1 mRNA and protein was significantly higher in HCC tissue and negatively correlated with survival. Neutrophils, Th17 cells, several immune inhibitors, and signaling pathways were positively correlated with the expression of HMGA1. Amplification was the main type of genetic alteration in HMGA1. These findings demonstrate that HMGA1 can be a therapeutic target and a potential biomarker to predict the prognosis of patients with HCC. HMGA1 may affect the progression of HCC by suppressing the immune function of these patients.

## 1. Introduction

Hepatocellular carcinoma (HCC) is the most common type of liver cancer and is the 4th leading cause of cancer-related death worldwide; thus, it has become a serious medical problem.^[1]^ Currently, surgical therapies, transarterial therapies, tumor ablation and systemic therapies are the majority of the clinical management. Among them, surgical resection is recognized as the most effective therapy for patients with early-stage HCC. However, HCC is often detected at an advanced stage, when surgery is often no longer an option. Sorafenib and regorafenib alone or combined with transarterial therapies have been recommended for patients at an advanced stage (BCLC stage B or C) since 2007.^[2,3]^ Nevertheless, the survival rate of patients with HCC is significantly lower than those with breast cancer, stomach cancer, esophageal cancer and intestinal cancer. In addition, the improvement in the survival rate is also significantly lower than that of those cancers in the past decade. Of note, immune-based therapies, such PD-1 inhibitors, have manifested clinical benefits for patients with HCC since 2017.^[3,4]^ Immune checkpoint inhibition is closely related to the critical role of the microenvironment in the progression of solid tumors, with immune activation occurring in 30% of patients with HCC at an early stage.^[3,5]^ Therefore, there is an urgent demand for identifying a new immune-based biomarker as a therapeutic target to improve the outcomes of patients with HCC.

The high mobility group A1 (HMGA1) gene is located on chromosome 6p21, with a molecular weight of 10 kDa.^[6]^ There is a lack of intrinsic transcriptional activity in HMGA1. However, HMGA1 protein is a nonhistone protein, and it can regulate chromatin architectural transcription factors, cell differentiation, tumorigenesis and adaptive immune responses.^[7,8]^ Previous studies have confirmed that there is an elevated expression level of HMGA1 in almost all human cancers, including breast cancer, neck cancer, thoracic cancer, abdominal cancer and reproductive system cancer.^[6,9]^ HMGA1 expression also correlates with metastasis and poor survival by targeting downstream genes such as IFN-*β*, IL-2 and IL-2R*α* or participates in many biological pathways, including TNF*α*/NF-*κ*B, EGFR, Hippo and Ras/ERK in thoracic cancer and Wnt/*β*-catenin and PI3K/Akt in abdominal cancer.^[6]^ Additionally, HMGA1 has been reported to modulate the tumor immune microenvironment.^[10]^ Moreover, PD-L1 could directly interact with HMGA1, and the upregulation of PD-L1 could activate HMGA1-dependent pathways and promote the expansion of colorectal cancer.^[11]^ However, only 1 existing study has focused on its role in antitumor immunity, which is not enough to support the results.^[12]^ The objective of this study was to analyze the expression of HMGA1 in HCC and its clinical significance, and the research methods used referred to previous studies on single genes.^[13]^ The expression of HMGA1 was determined based on data from public databases. In addition, the correlation between HMGA1 and prognosis was explored. Moreover, the tumor-infiltrating immune cells and the expression of immunosuppressive genes were also investigated. These results demonstrated a potential target for treating HCC.

## 2. Methods

### 2.1. HMGA1 gene and protein expression analysis

Public databases do not include case identification information, so patient consent and ethics approval were not needed. The expression levels of HMGA1 mRNA in HCC and normal liver tissue samples were collected from the cancer genome atlas (TCGA) database and the GSE10143,^[14]^ GSE14520^[15]^ and GSE36376^[16]^ datasets in the gene expression omnibus database. Subsequently, the expression levels of HMGA1 in the different datasets were detected. The HMGA1 protein expression levels in normal liver tissue and HCC samples were retrieved from the Human Protein Atlas database.^[17]^

### 2.2. Survival analysis

The correlation between HMGA1 expression and HCC patients’ clinical outcomes was explored on the basis of the Kaplan–Meier (KM) Plotter database.^[18]^ Data for a total of 371 patients were recorded in this database. The prognostic value of HMGA1 was determined based on progression-free survival (PFS), recurrence-free survival, disease-specific survival and overall survival (OS). In addition, univariate and multivariate prognostic analyses were conducted to explore the value of different clinicopathological factors and HMGA1 as predictors.

### 2.3. Gene set cancer analysis analysis (GSCA) and TISIDB analysis

The GSCA database integrates over 10,000 multidimensional genomic data from TCGA and can be used for immunogenomic cancer analysis.^[19]^ The correlations between HMGA1 gene expression and the proportions of 24 immune cell types were analyzed with this database. The TISIDB database contains information on 988 genes involved in antitumor immunity. These data were collected from the literature, high-throughput screening data, immunotherapy cohort datasets and public databases, including the TCGA and gene ontology database.^[20]^ TISIDB can be employed to analyze the interaction between the selected genes and immune-related genes. Therefore, several immunoinhibitors correlated with HMGA1 were explored based on the TISIDB database. Changes in immune cells and immunoinhibitors after the methylation of HMGA1 were also investigated.

### 2.4. Pathway identification

In this study, gene set enrichment analysis (GSEA) was used to identify specific pathways related to the high expression of HMGA1. This analysis method can compare the selected genes with predefined gene sets. By analyzing the data of gene expression profiles, their expression levels in specific functional gene sets can be revealed. Pathways with NES > 2 and FDR < 0.001 were considered significantly enriched functional pathways.

### 2.5. cBioPortal database analysis

The cBioPortal for Cancer Genomics is a tool for exploring multidimensional cancer genomics datasets.^[21]^ In this study, this tool was adopted to analyze the mutation, copy number variation (CNV), and gene cooccurrence of HMGA1.

### 2.6. Statistical analysis

**Figure 1.**
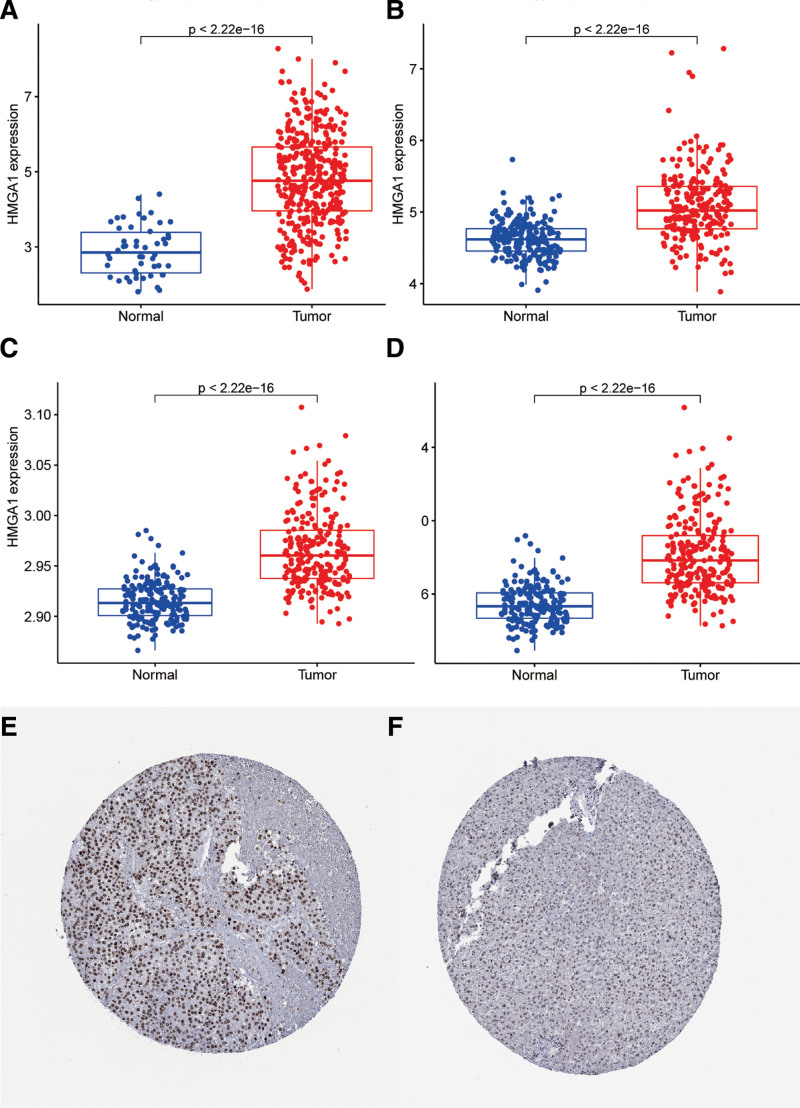
HMGA1 expression levels in HCC and normal liver tissues. Data were obtained from the (A) TCGA database and (B) GSE10143, (C) GSE14520 and (D) GSE36376 datasets. (E-F) Expression in tissues was analyzed by immunohistochemistry. The scale bar is 200 *μ*m. HCC = hepatocellular carcinoma, HMGA1 = the high mobility group A1, TCGA = the cancer genome atlas.

Data sources refer to (see Table S1, Supplemental Digital Content, http://links.lww.com/MD/I344, which provides the data sources for each figure). R software (version 4.04, http://www.r-project.org/) was used to perform the statistical analyses (see Table S2, Supplemental Digital Content, http://links.lww.com/MD/I345, which provides R software codes for Fig. 1A–D, Figs. 3 and 6). The rank sum test was conducted to analyze the difference among groups. The best performing threshold is used as the cutoff in the KM Plotter. Gene set enrichment analysis was conducted with the GSEA base and clusterProfiler packages. *P* < .05 in the 2-sided tests was considered to indicate statistical significance.

**Figure 2.**
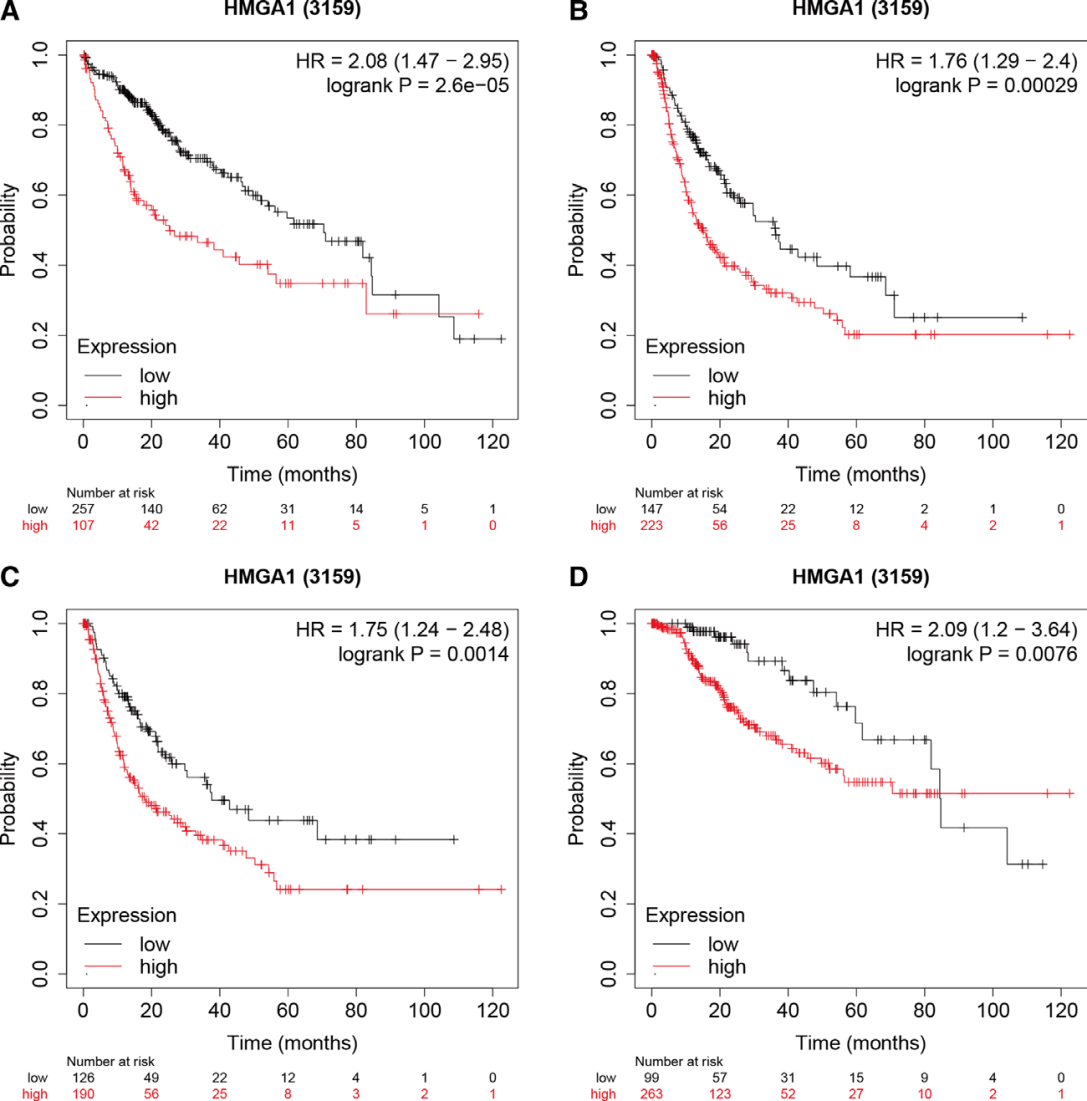
Correlation between the expression of HMGA1 and patient prognosis. (A–D) High HMGA1 expression correlates with worse PFS, RFS, DSS and OS. The number of patients who were at risk is shown below the figures. DSS = disease-specific survival, HMGA1 = the high mobility group A1, OS = overall survival, PFS = progression-free survival, RFS = recurrence-free survival.

**Figure 3.**
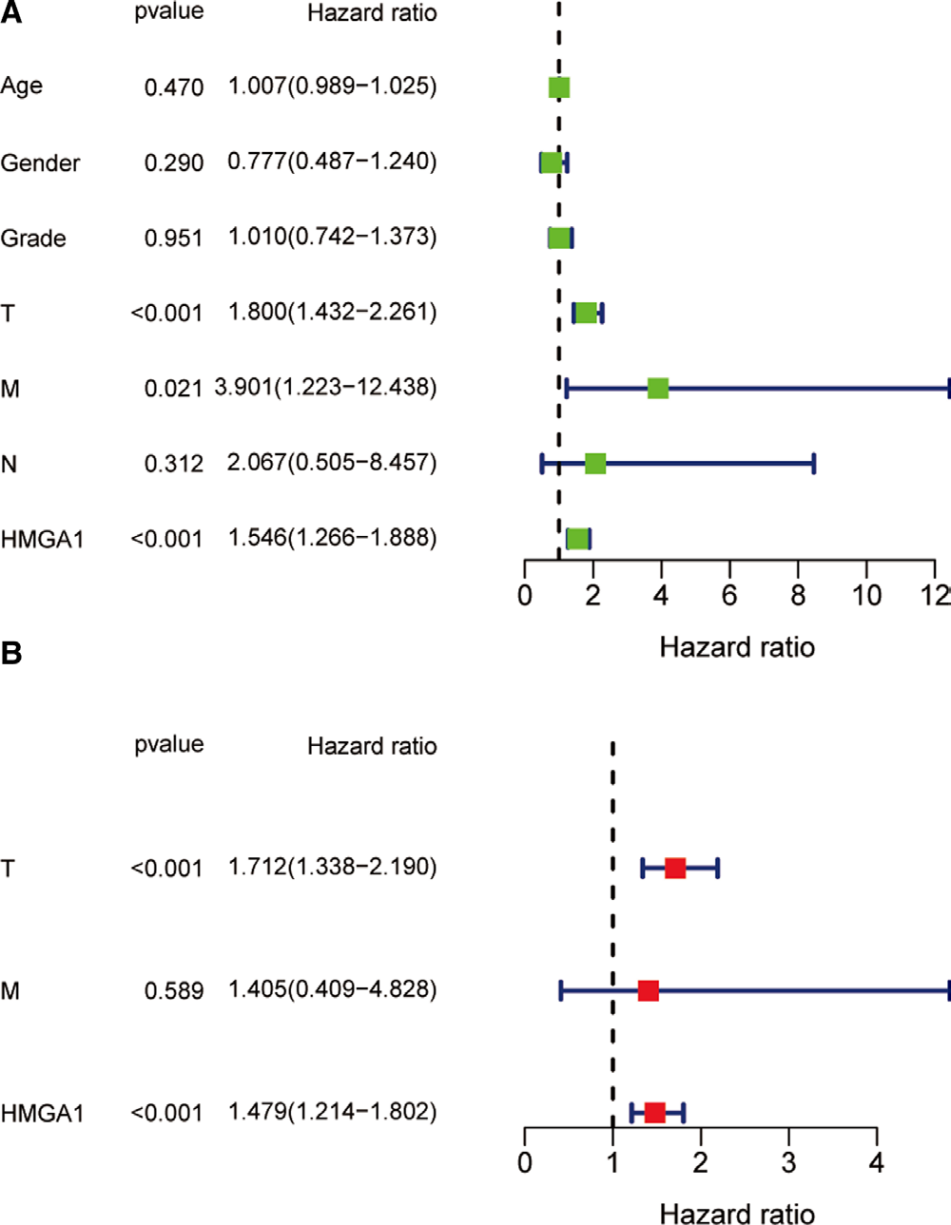
(A) Univariate and (B) multivariate prognostic analyses. HMGA1 and clinicopathological factors were included in the analysis. HMGA1 = the high mobility group A1.

**Figure 4.**
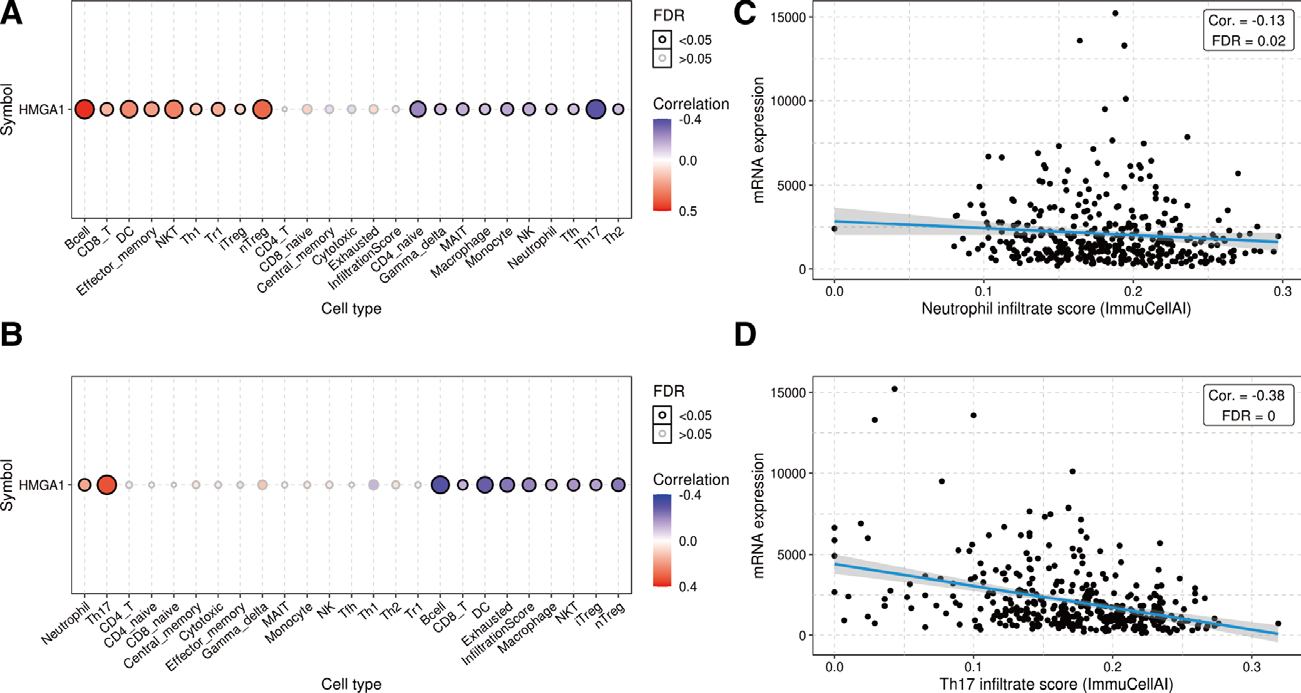
Correlation between the expression of HMGA1 and immune cells. (A–B) Comparison of immune cells before and after HMGA1 methylation. (C–D) The infiltration scores of neutrophils and Th17 cells are negatively correlated with HMGA1 expression. HMGA1 = the high mobility group A1.

**Figure 5.**
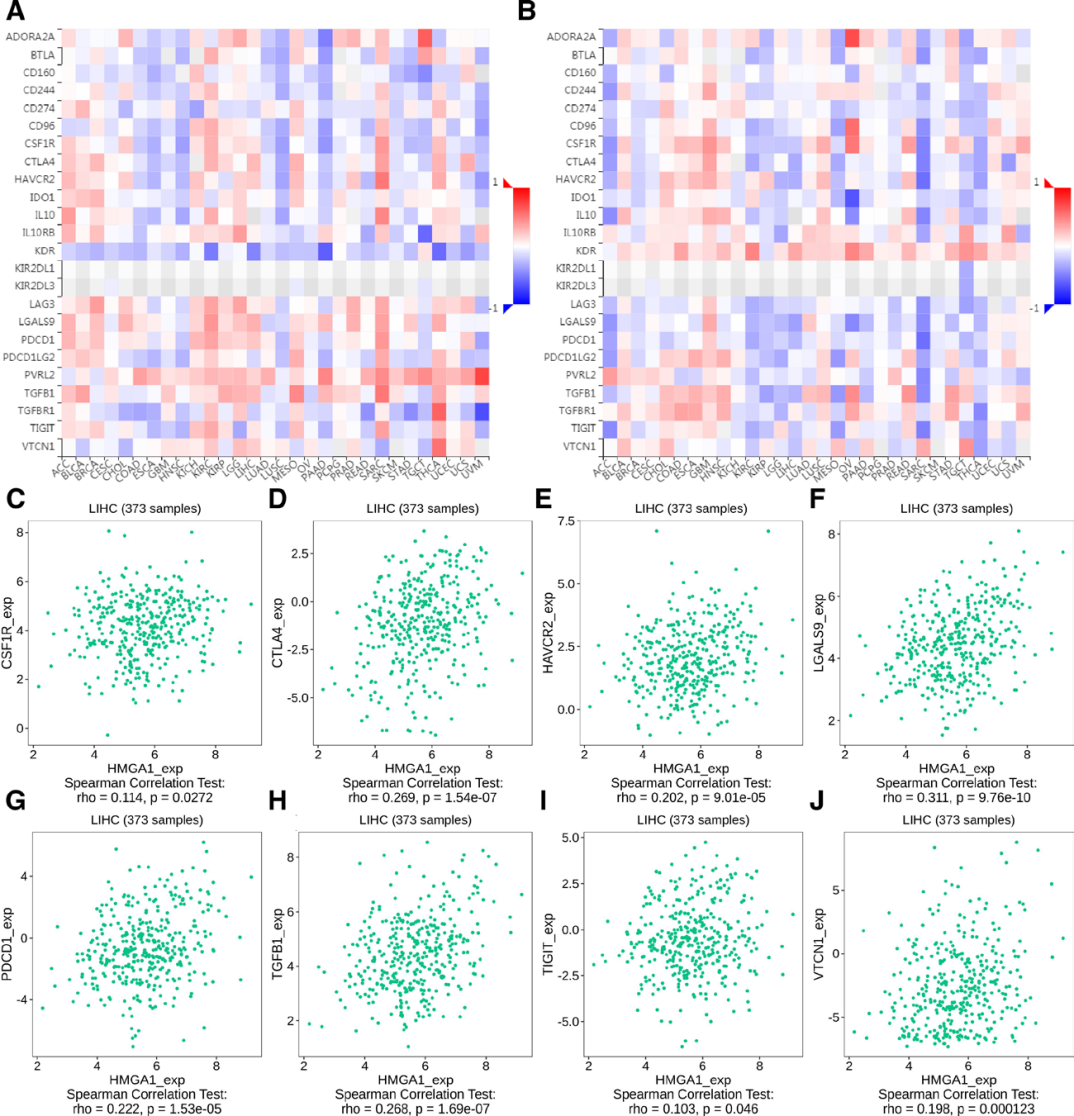
Correlation between the expression of HMGA1 and immunoinhibitors. (A–B) Comparison of immune inhibitor expression before and after HMGA1 methylation. (C–J) Correlations between HMGA1 and (C) CSF1R, (D) CTLA4, (E) HAVCR2, (F) LGALS9, (G) PDCD1, (H) TGFB1, (I) TIGIT and (J) VTCN1. HMGA1 = the high mobility group A1.

## 3. Results

### 3.1. The expression levels of HMGA1 mRNA and protein were increased in HCC

These data from the TCGA database showed that HMGA1 mRNA was highly expressed in HCC tissue compared with normal liver tissue (Fig. 1A), which was consistent with the results from 3 gene expression omnibus datasets (Fig. 1B–D). The results also revealed that the levels of HMGA1 protein in HCC tissue were 75% higher, with a higher staining rate and stronger intensity than those in normal liver tissues (Fig. 1E–F). These findings indicated that HMGA1 was overexpressed in HCC tissue.

### 3.2. A higher level of HMGA1 is related to a poor prognosis

The clinicopathological factors of patients used for KM analysis subgrouping are listed in Table 1. The higher expression level of HMGA1 was demonstrated to be related to the prognosis of patients with HCC based on the KM Plotter, including PFS, recurrence-free survival, disease-specific survival and OS (Fig. 2). Univariate and multivariate analyses indicated that both HMGA1 expression and tumor T stage were independent predictive factors (Fig. 3), and the low expression of HMGA1 and early T stage implied a better prognosis. These 2 independent predictive factors can be employed to predict the prognosis of HCC patients.

**Table 1:** Correlation between The high mobility group A1 mRNA expression and prognosis in Hepatocellular carcinoma with different clinicopathological factors by Kaplan–Meier plotter.

Clinicopathological factors	N	Overall survival		Progression-free survival	
		Hazard ratio	*P* value	Hazard ratio	*P* value
Gender					
Male	250	2.63 (1.69–4.09)	< .001	1.89 (1.28–2.80)	.0012
Female	121	1.97 (1.09–3.54)	.02	1.81 (1.06–3.09)	.03
Stage					
1	171	2.35 (1.12–4.92)	.02	1.38 (0.83–2.30)	.22
2	86	3.76 (1.29–10.99)	.0092	2.18 (1.10–4.31)	.02
3	85	3.47 (1.88–6.41)	< .001	2.41 (1.39–4.20)	.0014
4	5	–	–	–	–
Grade					
1	55	2.49 (0.88–7.02)	.07	3.57 (1.51–8.49)	.0021
2	177	2.15 (1.23–3.74)	.0057	1.86 (1.17–2.95)	.0076
3	122	3.02 (1.63–5.63)	< .001	1.89 (1.02–3.49)	.04
4	12	–	–	–	–
AJCC_T					
1	181	2.38 (1.18–4.80)	.01	1.47 (0.89–2.43)	.13
2	94	2.80 (1.14–6.89)	.02	2.04 (1.09–3.84)	.02
3	80	3.03 (1.62–5.67)	< .001	2.02 (1.14–3.58)	.01
4	13	–	–	–	–
Vascular invasion					
Yes (micro)	93	2.40 (1.12–5.15)	.02	1.83 (0.98–3.42)	.05
Yes (macro)	16	–	–	–	–
None	205	2.71 (1.36–5.41)	.0034	1.82 (1.05–3.16)	.03
Race					
White	184	2.18 (1.3–3.66)	.0025	2.08 (1.33–3.23)	< .001
Asian	158	3.38 (1.86–6.13)	< .001	2.94 (1.54–5.60)	< .001
Black or African American	17	–	–	–	–
Alcohol consumption					
Yes	117	4.02 (2.11–7.65)	< .001	2.43 (1.38–4.29)	.0015
None	205	1.6 (1.00–2.55)	.05	1.54 (1.02–2.30)	.04
Virus hepatitis					
Yes	153	2.24 (1.17–4.29)	.01	1.32 (0.80–2.17)	.27
None	169	2.69 (1.65–4.40)	< .001	2.46 (1.59–3.82)	< .001

### 3.3. HMGA1 expression is correlated with immunosuppression

The GSCA results revealed the infiltration of immune cells before and after the methylation of HMGA1 (Fig. 4A–B). The expression of neutrophils and Th17 cells decreased when HMGA1 was overexpressed but increased when HMGA1 was methylated (Fig. 4C–D). These data showed that the expression of CSF1R, CTLA4, HAVCR2, LGALS9, PDCD1, TGFB1, TIGIT and VTCN1 increased when HMGA1 was positive but decreased after HMGA1 was methylated (Fig. 5). The GSCA and TISIDB results confirmed that HMGA1 correlated with the immunosuppressive microenvironment by affecting immune cells and immunosuppressive factors.

### 3.4. GSEA

**Figure 6.**
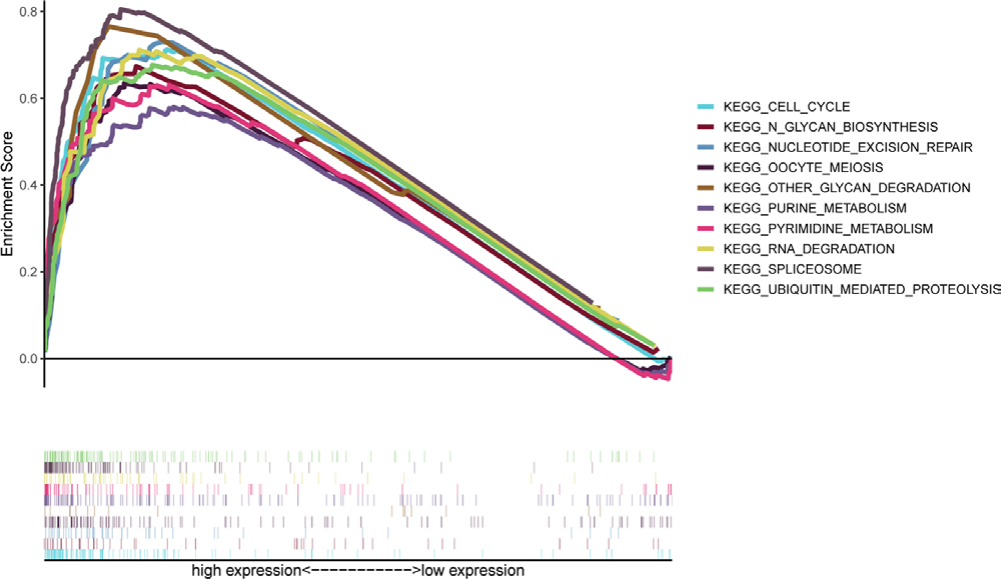
GSEA analyses of HMGA1. Samples were classified into high and low HMGA1 expression groups. The statistically significant results are shown. GSEA = gene set enrichment analysis, HMGA1 = the high mobility group A1.

To identify the specific gene signatures related to HMGA1, TCGA RNA-Seq data of liver hepatocellular carcinoma were subjected to an analysis of differential gene expression. The carcinogenesis and tumor progression pathways regulated by HMGA1 were explored. The GSEA results revealed that HMGA1 expression was positively correlated with several pathways, including “cell cycle,” “glycan biosynthesis,” “nucleotide excision repair,” “meiosis,” “other glycan degradation,” “purine metabolism,” “pyrimidine metabolism,” “RNA degradation,” “spliceosome” and “ubiquitin-mediated proteolysis” (Fig. 6).

### 3.5. Genomic alterations of HMGA1

**Figure 7.**
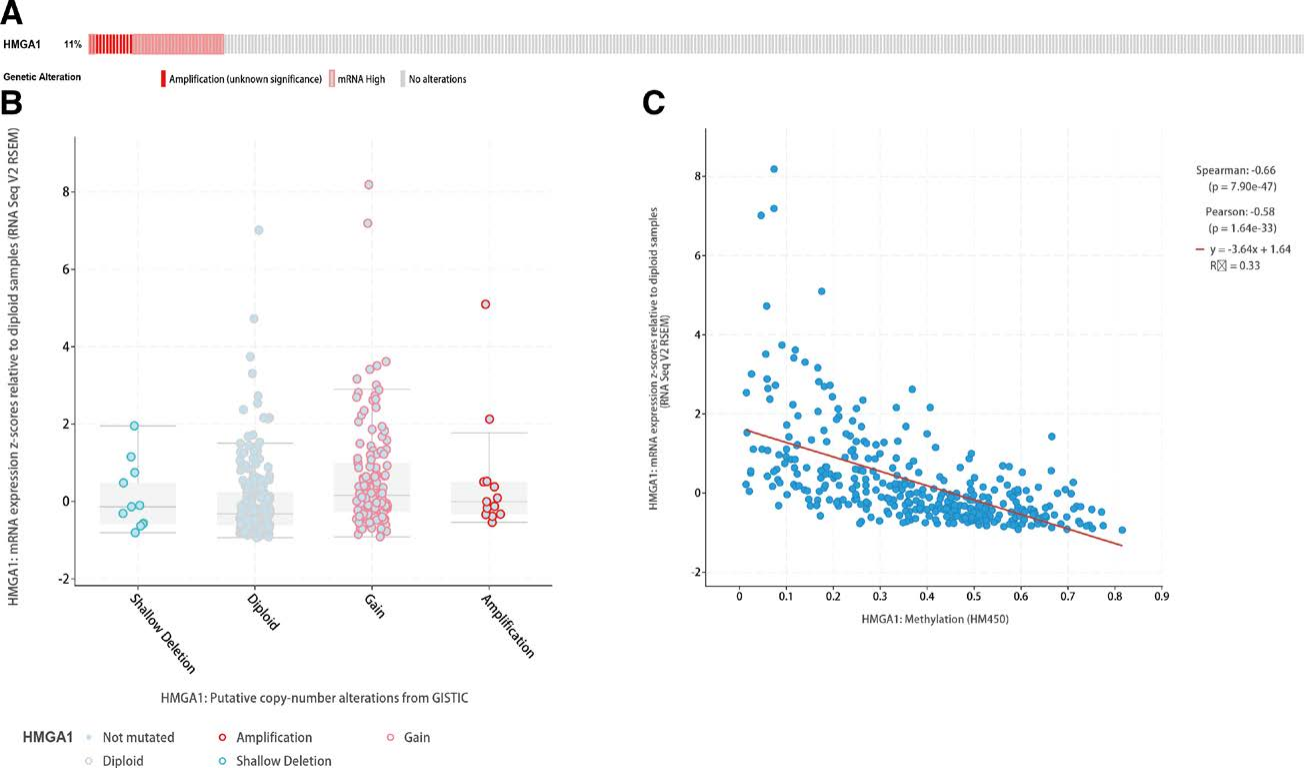
Genomic alterations of HMGA1 in the LIHC cohort. (A) OncoPrint of HMGA1 alterations. (B) HMGA1 expression in the 4 CNV groups. (C) Relation between the methylation of HMGA1 and its expression level. CNV = copy number variation. HMGA1 = the high mobility group A1, LIHC = liver hepatocellular carcinoma.

The analyses of genomic alterations of HMGA1 based on cBioPortal indicated that 40 (11%) out of 360 patients with HCC had alterations in HMGA1 (Fig. 7A). These alterations included 7% mRNA upregulation (n = 25) and 4% amplification (AMP) (n = 15). Because there was no other alteration type, AMP was the major type of HMGA1 CNV in HCC. The amplification of HMGA1 resulted in high expression of HMGA1 (Fig. 7B), while HMGA1 methylation indicated low expression of HMGA1 (Fig. 7C).

## 4. Discussion

HCC is 1 of the most common and fatal cancers, and the existing therapeutic regimens are not effective enough, which leads to a persistently high mortality rate in HCC patients. Therefore, the exploration of the potential therapeutic targets of HCC is of great significance for determining new and efficient therapeutic methods. As a defense system of the human body, the immune system fulfills a crucial function in the treatment of cancer. Cancer cells can be eliminated by strengthening immune function or modifying immune response molecules. Although immunotherapy provides a possibility for the treatment of patients with HCC, its efficacy is still not fully confirmed in existing studies, and it often induces serious side effects. Therefore, the exploration of these genes, which are highly expressed in HCC tissue and potentially related to immunity, is of great value for the development of immunotherapy.

The HMGA1 proteins in mammals function as structural transcription factors, and they can participate in the activation of many target gene promoters and are related to several cellular processes. Under normal conditions, the expression of HMGA1 only occurs at the embryogenesis stage and decreases with the progression of organogenesis. The expression of HMGA1 can hardly be detected in mature cells.^[6]^ In recent years, high expression of HMGA1 has been found in many cancers, and its expression positively correlates with the degree of malignancy and metastasis of tumors.^[22,23]^ Silencing HMGA1 can block the metastasis of breast cancer and colorectal cancer and reduce the proportion of tumor stem cells.^[24–26]^ As reported in a recent study, the high expression of HMGA1 may be induced by the secretion of breast cancer cells, and signals may be sent through the advanced glycation end-products receptor, which would promote invasiveness.^[26]^ In addition, previous studies demonstrated that the overexpression of HMGA1 can regulate the epigenetic silencing of genes related to tumor progression. Therefore, inhibiting HMGA1 may be effective in treating HCC.^[12]^

This study was conducted on the basis of data from a large sample database, with the aim of analyzing the expression of HMGA1 in HCC tissue and its clinical significance. In this study, we found that the levels of HMGA1 mRNA and protein in HCC tissue were significantly higher than those demonstrated to correlate with a poor prognosis, and almost all patients with HCC had poor OS and PFS, regardless of their clinical features. In addition, the results of univariate and multivariate analyses reveal that HMGA1 and T stage are independent prognostic factors in patients with HCC. According to a previous study, HMGA1 is related to immune responses. For instance, HMGA1 can regulate the expression of IFN-*γ*, which is mainly produced by CD8 + T cells, CD4 + T cells and NK cells.^[27]^ However, the influence of increases in HMGA1 on the immunity of patients with HCC remains unclear. As per the findings in this study, the expression of HMGA1 positively correlates with the immunosuppressive microenvironment. HMGA1 reduced the expression of neutrophils and Th17 cells in patients with HCC. Neutrophils could play key roles in regulating HCC progression, while Th17 cells could participate in the inhibition of the invasion and progression of HCC. Furthermore, these 2 cell types are regulated by IFN-*γ*.^[28,29]^ Hence, HMGA1 may directly inhibit the production of neutrophils and Th17 cells or may indirectly inhibit their production by inhibiting the production of IFN-*γ*. Moreover, HMGA1 positively correlates with several immune inhibitors (CSF1R,^[30]^ CTLA4,^[31]^ HAVCR2,^[32]^ LGALS9,^[33]^ PDCD1,^[34]^ TGFB1,^[35]^ TIGIT^[36]^ and VTCN^[37]^), all of which have been demonstrated as potential candidates for immunotherapy. Methylation makes genes silent. Here, we demonstrated that the exact effect of HMGA1 on immune cells from both positive and negative aspects. When HMGA1 is methylated, the expression of both immune cells is upregulated, and that of immune inhibitors is downregulated. Thus, the findings of this study provide a new line of evidence for the correlation of HMGA1 with immunosuppression and poor prognosis. The GSEA results suggested that HMGA1 correlates with multiple signaling pathways. As confirmed in a previous study, the cell cycle is a critical driver and a key biological process in HCC.^[38]^ Nucleotide excision repair, pyrimidine metabolism, spliceosome, glycan degradation, and RNA degradation have also been reported to correlate with carcinogenesis and progression in various cancers.^[39–41]^ Genomic alterations of HMGA1 showed that AMP was the major type of HMGA1 CNV in HCC. Gene amplification is an increase in the number of copies of a gene sequence. Cancer cells sometimes produce multiple copies of genes in response to signals from other cells or their environment. The main types: amplification, gain, deep deletion, and shallow deletion are derived from copy-number analysis algorithms, and indicate the copy-number level per gene. These calls are putative and the deep deletions and amplifications are biologically relevant to individual genes by default. Based on these results, it can be proposed that HMGA1 inhibition alone or in combination with immune checkpoint inhibitors may be beneficial to patients with HCC.

Although these findings provide a new method for HCC immunotherapy, there are still some limitations to this study. The clinical significance of HMGA1 in HCC was analyzed based on public databases. Although the sample size is large and the samples have favorable homogeneity, it is difficult to avoid the inherent bias of retrospective studies. In this study, the clinical samples were not collected and tested to verify these results. Moreover, it is necessary to conduct cell and animal experiments to confirm the role of HMGA1 in immune cell infiltration and HCC progression.

In conclusion, these results demonstrate that HMGA1 is a significant adverse prognostic biomarker that participates in immunosuppression in HCC. HMGA1 may affect tumor progression by suppressing the infiltration of neutrophils and Th17 cells, promoting the expression of immune inhibitors and interacting with other signaling pathways. However, further studies are required to validate the relevant conclusions.

## Acknowledgements

The authors thanks for the data support from included studies.

## Author contributions

**Conceptualization:** Jie Zhu, Yongshun Zheng, Yuyao Liu, Mengding Chen, Yanyan Liu, Jiabin Li.

**Data curation:** Jie Zhu, Yuyao Liu.

**Formal analysis:** Jie Zhu, Yongshun Zheng.

**Funding acquisition:** Jiabin Li.

**Investigation:** Jie Zhu, Yongshun Zheng, Yuyao Liu, Mengding Chen.

**Methodology:** Jie Zhu.

**Project administration:** Yanyan Liu, Jiabin Li.

**Resources:** Jie Zhu, Yongshun Zheng, Yuyao Liu.

**Software:** Yongshun Zheng, Yuyao Liu, Mengding Chen.

**Supervision:** Yanyan Liu, Jiabin Li.

**Validation:** Yongshun Zheng, Yuyao Liu, Mengding Chen.

**Visualization:** Jie Zhu, Yongshun Zheng.

**Writing – original draft:** Jie Zhu.

**Writing – review & editing:** Mengding Chen, Yanyan Liu, Jiabin Li.

## Supplementary Material
