# Supplementary material for: Association between HMGA1 and immunosuppression in hepatocellular carcinoma: A comprehensive bioinformatics analysis
Source: Medicine (Baltimore). 2023 Jan 27;102(4):e32707. doi: 10.1097/MD.0000000000032707 (PMC9876027; doi:10.1097/MD.0000000000032707)
Supplement: Supplementary file 1 [file medi-102-e32707-s001.pdf]

| | Source data | Website |
|---|---|---|
| Table 1, Figure 1 A-D | GEO, EGA, and TCGA | https://kmplot.com/analysis/ |
| Citation: Lanczky A, Gyorffy B: Web-Based Survival Analysis Tool Tailored for Medical Research (KMplot): Development and Implementation, J Med Internet Res, 2021 Jul 26;23(7):e27633. doi: 10.2196/27633 | | |
| Figure 1 E-F | TCGA | http://www.proteinatlas.org |
| Citation: Uhlen M, Fagerberg L, Hallstrom BM, Lindskog C, Oksvold P, Mardinoglu A, et al. Proteomics. Tissue-based map of the human proteome. Science. 2015;347(6220):1260419. | | |
| Figure 2,3 | GEO, EGA, and TCGA | https://kmplot.com/analysis/ |
| Citation: Lanczky A, Gyorffy B: Web-Based Survival Analysis Tool Tailored for Medical Research (KMplot): Development and Implementation, J Med Internet Res, 2021 Jul 26;23(7):e27633. doi: 10.2196/27633 | | |
| Figure 4 | TCGA | http://bioinfo.life.hust.edu.cn/GSCA/#/immune |
| Citation: Liu CJ, Hu FF, Xia M, Han L, Zhang Q, Guo AY. (2018). GSCALite: A Web Server for Gene Set Cancer Analysis. Bioinformatics. | | |
| Figure 5,6 | TCGA | http://cis.hku.hk/TISIDB/index.php |
| Citation: Beibei Ru, Ching Ngar Wong, Yin Tong, Jia Yi Zhong, Sophia Shek Wa Zhong, Wai Chung Wu, Ka Chi Chu, Choi Yiu Wong, Chit Ying Lau, Ian Chen, Nam Wai Chan, Jiangwen Zhang. TISIDB: an integrated repository portal for tumor-immune system interactions. Bioinformatics. 2019; btz210. | | |
| Figure 7 | TCGA | http://cbioportal.org |
| Citation: Sumter TF, Xian L, Huso T, Koo M, Chang YT, Almasri TN, et al. The High Mobility Group A1 (HMGA1) Transcriptome in Cancer and Development. Current molecular medicine. 2016;16(4):353-93. | | |
